# Measurement method of tear meniscus height based on deep learning

**DOI:** 10.3389/fmed.2023.1126754

**Published:** 2023-02-14

**Authors:** Cheng Wan, Rongrong Hua, Ping Guo, Peijie Lin, Jiantao Wang, Weihua Yang, Xiangqian Hong

**Affiliations:** ^1^College of Electronic Information Engineering, Nanjing University of Aeronautics and Astronautics, Nanjing, China; ^2^Shenzhen Eye Hospital, Jinan University, Shenzhen, China; ^3^Shenzhen Eye Institute, Shenzhen, China

**Keywords:** tear meniscus height, dry eye disease, automatic diagnosis, deep learning, image segmentation

## Abstract

Tear meniscus height (TMH) is an important reference parameter in the diagnosis of dry eye disease. However, most traditional methods of measuring TMH are manual or semi-automatic, which causes the measurement of TMH to be prone to the influence of subjective factors, time consuming, and laborious. To solve these problems, a segmentation algorithm based on deep learning and image processing was proposed to realize the automatic measurement of TMH. To accurately segment the tear meniscus region, the segmentation algorithm designed in this study is based on the DeepLabv3 architecture and combines the partial structure of the ResNet50, GoogleNet, and FCN networks for further improvements. A total of 305 ocular surface images were used in this study, which were divided into training and testing sets. The training set was used to train the network model, and the testing set was used to evaluate the model performance. In the experiment, for tear meniscus segmentation, the average intersection over union was 0.896, the dice coefficient was 0.884, and the sensitivity was 0.877. For the central ring of corneal projection ring segmentation, the average intersection over union was 0.932, the dice coefficient was 0.926, and the sensitivity was 0.947. According to the evaluation index comparison, the segmentation model used in this study was superior to the existing model. Finally, the measurement outcome of TMH of the testing set using the proposed method was compared with manual measurement results. All measurement results were directly compared via linear regression; the regression line was y0.98x−0.02, and the overall correlation coefficient was *r*^2^0.94. Thus, the proposed method for measuring TMH in this paper is highly consistent with manual measurement and can realize the automatic measurement of TMH and assist clinicians in the diagnosis of dry eye disease.

## 1. Introduction

Dry eye disease (DED) is a multifactorial disease of the ocular surface that is accompanied by increased tear film osmolality and ocular surface inflammation, causing symptoms such as visual impairment and tear film instability (1, 2) and potential damage to the ocular surface, affecting the visual function of millions of people worldwide. In traditional diagnostic methods for DED, Schirmer’s test, tear break-up time measurement, and ocular surface staining score are commonly used to qualitatively and quantitatively analyze the tear film (3, 4). Tear meniscus height (TMH) can be used to assess the tear volume and tear film status. The tear meniscus is located at the edge of the upper and lower eyelids and accounts for 75–90% of the total tear volume (5). The lower tear meniscus is more stable, and the DED analysis mainly uses the lower tear meniscus index, which is also aimed at the lower tear meniscus. Previous studies have reported decreased tear meniscus parameters (TMH, tear meniscus volume, and tear meniscus dynamics) in DED patients (6–8). Therefore, the quantification of tear meniscus parameters is helpful in the diagnosis of DED. As a crucial parameter of the tear meniscus, the TMH has received extensive attention in recent years. In fact, in current clinical studies, although screening of the tear meniscus is performed by non-contact eye photography, the quantitative measurement of TMH is mostly manual or semi-automated. For example, physicians need to be involved in the assessment process of identifying and outlining the upper and lower edges of the tear meniscus in the image, and the measurement points of the TMH are empirically selected by the physician. These subjective assessments may lead to inconsistent results, reduced repeatability, and increased interobserver variability (9, 10). Manual measurement of TMH is time consuming and laborious if a large number of images are involved.

As a crucial parameter of tear meniscus, TMH has received increasing attention in recent years, and screening for DED can be achieved by assessing TMH. Stegmann et al. (11) assessed TMH, tear meniscus area, tear meniscus depth, and tear meniscus radius using image data acquired by ultra-high resolution optical coherence tomography combined with conventional image processing algorithms. In 2019, Yang et al. (12) from the Human Research and Ethics Committee of Peking University Third Hospital implemented a brand-new automated tear meniscus segmentation and height measurement software ImageJ based on a multi-threshold segmentation algorithm and compared the ImageJ measurement results with the manual measurement results. Arita et al. (13) successfully segmented and measured the tear meniscus by interference fringes using a DR-1α tear interferometer and achieved high accuracy. However, this method is not fully automated and requires manual selection of the measurement point. In 2020, Stegmann et al. (14) improved the threshold-based segmentation algorithm to a convolutional neural network segmentation algorithm based on (11) and found that the use of deep learning segmentation algorithm increased the operation speed by 228 times compared with threshold segmentation algorithm. In 2021, researchers from the School of Biomedical Engineering, Department of Medicine, Shenzhen University (15) proposed a tear meniscus segmentation algorithm based on a fully convolutional neural network and combined with polynomial fitting of the upper and lower edges of the tear meniscus to measure TMH, and the measurement results of the TMH were compared with the manual measurement results. However, it was easy to deviate when the tear meniscus edge was fitted with the polynomial, and polynomial fitting was required for each measurement of a picture.

There are two main findings of this study. First, combined with the existing segmentation network structure and the characteristics of ocular surface images, a segmentation network suitable for this experiment is built to accurately segment the tear meniscus region and the central ring of the corneal projection ring (CCPR). Thereafter, combined with the image processing method, the center point of the CCPR is located, and the region that needs to be evaluated for the TMH is selected. Finally, the TMH measurement method is continuously adjusted, and the final measurement method is determined. The processes of ocular surface image acquisition, tear meniscus region segmentation, and TMH measurement are fully automatic and noninvasive (16, 17). In addition, we compared the measured results of TMH using the method proposed in this study with those of experienced professional doctors to evaluate the feasibility of the proposed method.

## 2. Dataset

**FIGURE 1:**
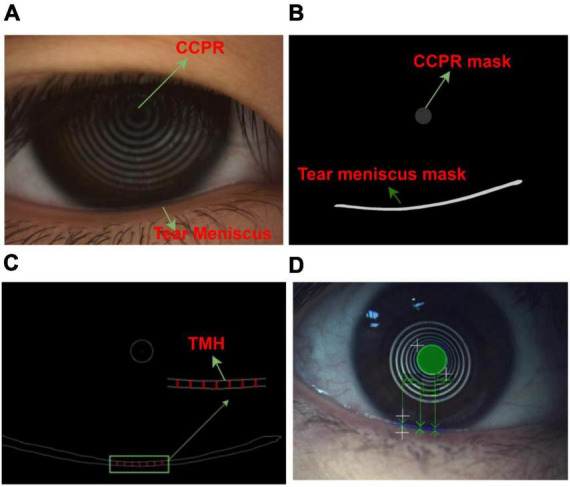
Ocular surface image data **(A)** Original ocular surface image **(B)** Segmentation convolution neural network segmented tear meniscus region and central ring of corneal projection ring (CCPR) region **(C)** Schematic of selected TMH measurement region **(D)** Schematic of physician assessment of TMH.

In total, 325 ocular surface images were obtained. All images were obtained from the Shenzhen Eye Hospital. All the data used in this experiment explained the purpose and possible results to the providers. All ocular surface images were acquired using a Keratograph 5 M (K5 M), during which the patients needed to place their chin on a stand in front of the K5 M, adjust the measured eye to a distance of 100 mm from the camera, face the camera, and remain still at the physician’s instructions. The images that caused unclear shooting due to unfocusing and closing eyes in 325 ocular surface images were manually eliminated. Finally, 305 clear images were obtained for the experiment. All images used for the experiment were in png format of 1,360 pixel × 1,024 pixel, as shown in Figure 1A. Furthermore, 305 ocular surface images were divided into training and testing sets, in which 270 ocular surface images were included in the training set, and 35 ocular surface images were included in the testing set. The training set is the data sample used for model fitting that performs gradient descent of training error during training and learns the trainable weight parameters. The testing set was a separate set of samples left during model training, which could be used to evaluate the performance of the model.

In the network training process, it is necessary to input the ocular surface image and its corresponding labeling image. All ocular surface images were labeled by a professional DED diagnostician. In the labeling process, time is not limited, and the edge of the tear meniscus region is accurately labeled to the extent possible. The labeled data are transformed into binary images, in which the labeled target region is represented by a pixel value of 1, and the background region is represented by a pixel value of 0, as shown in Figure 1B. To measure TMH, we selected several measuring points, as shown in Figure 1C, the physician selected several measuring points in the tear meniscus region near the right underneath corresponding to the center point of the CCPR, assessed TMH at these measuring points, and subsequently averaged them as the final TMH measurement, as shown in Figure 1D.

## 3. Materials and methods

**FIGURE 2:**
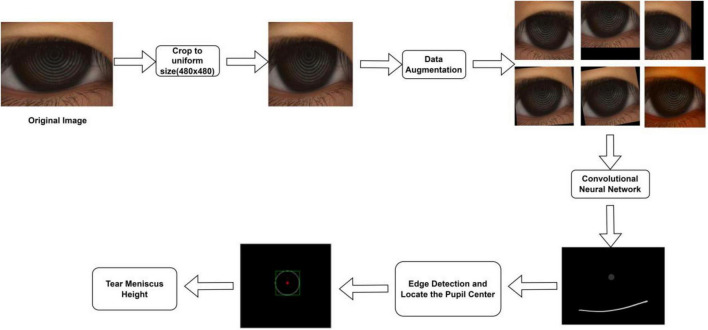
Automatic measurement process of TMH.

In this study, the TMH was evaluated using the following steps: (1) The original ocular surface image and its corresponding tear meniscus region labeling mask in the training set were preprocessed, data augmentation was performed, the processed data were sent into the deep convolution neural network for network training, and the network parameters of the optimal model were saved. (2) The original ocular surface images and the CCPR in the training set were labeled with a mask for preprocessing and data augmentation. The processed data were fed into a deep convolution neural network for network training, and the network parameters of the optimal model were stored. (3) Load the network weights obtained in (1) and (2) to predict the tear meniscus region and the CCPR region of the ocular surface image in the testing set, respectively. (4) The center of CCPR was located. In this study, the center of the CCPR was located by considering the pixel coordinates and edge lengths of the upper left corner of the rectangle through an external rectangle of the predicted CCPR that requires circular fitting of the predicted CCPR to achieve a more accurate center position. (5) Prediction of TMH. The main flow of tear meniscus segmentation and height measurement is shown in Figure 2.

### 3.1. Segmentation model structure

**FIGURE 3:**
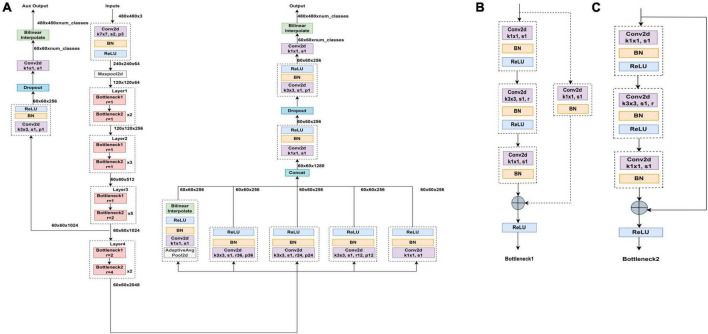
Segmented network structure diagram **(A)** Segmented network structure is composed of resnet50 module, ASPP module, and upper sampling. Panels **(B,C)** are bottleneck1 and bottleneck2, respectively, all of which are composed of several convolutional layers, batch normalization and ReLU.

To achieve segmentation of the tear meniscus region, we built a deep convolutional neural network based on the DeepLabv3 (18) architecture, which was initially used for semantic segmentation. To segment the tear meniscus region better, the DeepLabv3 network was adjusted and improved in this study. The segmentation of the tear meniscus region is performed through feature extraction and image size restoration to obtain the final segmentation results, which include the backbone module and ASPP (19) module. The backbone module used in this study refers to resnet50 (20) and renders certain improvements based on the characteristics of the tear meniscus image. The entire backbone consists of a 7 × 7 convolutional layer, maximum pooling layer, and four blocks. Each block consists of bottleneck1 and bottleneck2. Both bottleneck1 and bottleneck2 are residual blocks composed of several convolutional layers, linear normalization layers, rectified linear units, and shortcut branches. Bottlenboteck1 differs from bottleneck2 in that a convolutional kernel of 1 × 1 is added to the shortcut branch of bottleneck1 to reduce the dimension. The specific structures of bottleneck1 and bottleneck2 are shown in Figures 3B, C, respectively. Certain common convolutional layers in block3 and block4 are replaced by atrous convolutional layers (21), and the specific expansion coefficient setting is shown in Figure 3A. The ASPP module consists of five parallel branches, which are a convolutional layer of 1 × 1, three atrous convolutional layers of 3 × 3, and a global average pooling layer that can increase global context information (followed by a convolutional layer of 1 × 1, and subsequently, the size of the input is restored by bilinear interpolation); thereafter, the outputs of these five branches are concatenated along the channel direction, and finally the information is further fused by a convolutional layer of 1 × 1. In addition, for the three parallel atrous convolutional layers, we use the multi-grid strategy and experimentally found that in the experiments performed in this study, the best results are obtained when the multi-grid is set to (1, 1, 1). The structure of the entire network is illustrated in Figure 3.

**FIGURE 4:**
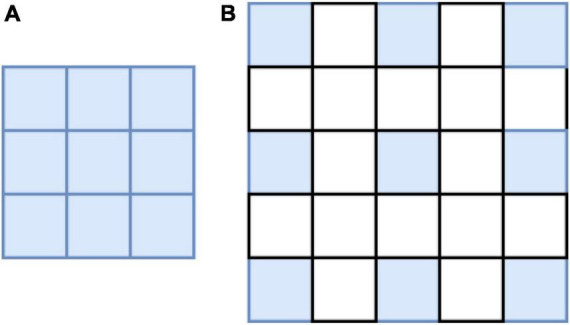
Convolutional kernel schematic: **(A)** Common convolution **(B)** atrous convolution.

In the process of feature extraction, the pooling and convolutional layers are generally used to increase the receptive field; however, this also reduces the size of the feature map. For segmentation, it is necessary to use upper sampling to restore the size of the feature map, and the process of feature map reduction and reamplification causes loss of accuracy. To solve this problem, the concept of atrous convolution, which can increase the receptive fields while maintaining the size of the feature maps is proposed. Atrous convolution introduces a hyperparameter called expansion rate, which defines the spacing of each value of the convolutional kernel when processing the data, as shown in Figure 4: (a) shows a common convolutional kernel with a size of 3 × 3; (b) shows an atrous convolution with a size of 5 × 5. The atrous convolutional kernel enlarges the size based on the ordinary convolutional kernel; however, the convolutional kernel unit that participates in the operation does not change; only the light blue square in the figure is the unit that participates in the operation, and the elements in the white square are filled with 0. Atrous convolution increases receptive fields by enlarging the size of the convolutional kernel, while neither increasing the computational load nor reducing the resolution of the feature map. The degree of expansion of the convolutional kernel can be controlled by the expansion factor, assuming that the expansion factor is S, the size of the common convolution kernel is K_0_, and the size of the convolution kernel after the expansion design is *K_c_*, as follows:


(1) $${\text{K}}_{c}=S\times \left(K_{0}-1\right)+1$$

In the process of restoring the image size, bilinear interpolation is adopted in this study. To further optimize the segmentation performance of the network, this study refers to the auxiliary classifier structure in GoogleNet (22) and the FCN (23) network structure, and the output of backbone’s block3 in the model leads to an FCN head as an auxiliary output.

At present, many segmentation networks are based on the improvement of Unet (24), which has also been widely used in the field of biomedical image segmentation, and this method was proposed at the MICCAI meeting in 2015 and has now reached more than 4000 citations. Unet is characterized by an encoder–decoder structure, and through the convolutional layer and pooling layer, the input picture information is encoded into the feature information that can be recognized by the computer, and subsequently, the compressed feature map is also sampled. Compared with the previous segmentation network, Unet fuses more low-level semantic information starting from the first convolutional layer, and the output feature maps of each layer are copied and concatenated with the decoded information after the subsequent upper sampling to generate a new feature map. In the last layer of the network, Unet uses a convolutional layer of 1 × 1 instead of the fully connected layer and uses a convolutional kernel of 1 × 1 to achieve dimensionality reduction, which is a linear transformation and superposition of the combination of information between different channels. Unet plays an important role in the segmentation of medical images owing to their lightweight network structure and feature concatenation. Therefore, to further evaluate the performance of the model used in this study, the Unet series network is selected as the experimental contrast model.

### 3.2. TMH measurement method

When assessing TMH, a professional doctor mainly evaluates the height of the tear meniscus region corresponding to the vicinity directly below the center of the CCPR, that is, several measurement points are selected in the tear meniscus region directly below the center of the CCPR for TMH assessment, and the average value of the assessment is the final TMH measurement result. Because each assessment requires the physician to select the assessment point, not only is it time consuming and labor intensive, but also the assessment results are susceptible to subjective factors. After consultation with experts in the field of DED diagnosis, this study used the method of averaging multiple measurement points, which is realized as follows: the pixel coordinate of the center of the CCPR is (x,y), the pixel coordinate corresponding to the upper edge of the tear meniscus is (x_i_,y_i_), and the pixel coordinates corresponding to the lower edge of the tear meniscus is (x_−i−1_,y_−i−1_). The pixel sets of |x_i_−x|<=100 and |x_−i−1_−x|<=100 are calculated. Because the edge of the tear meniscus includes the upper and lower edges, 400 pixel coordinates can be obtained. Referring to the opinions given by professional doctors, this study selects an upper tear river coordinate value(x_j_,y_j_) and its corresponding tear river coordinate value (x_−j−1_,y_−j−1_) every 30 pixels to calculate the TMH. In addition, a previous study (15) showed that TMH in the tear meniscus region 0.5–4 mm directly below the center of the CCPR has strong robustness, and the TMH value in this region is insensitive to the selected measurement points. Therefore, a total of seven TMH measurement points were selected in the tear meniscus region 2 mm directly below the center of the CCPR, and the pixel values corresponding to these seven TMHs were averaged to obtain the final TMH, as shown in Equation 2. The height and width of all pictures used during the experiment were measured and averaged, and this step was repeated three times to finally obtain the height and width of the ocular surface image as 11.85 mm and 15.75 mm, respectively, and subsequently, the pixel value could be converted to a height value by the conversion formula of Equation 3, as shown in Figure 1C.


(2) $$\text{PTMH}=\frac{1}{7}.\sum_{\text{j}=1}^{7}\left|{\text{y}}_{\text{j}}-{\text{y}}_{-\text{j}-1}\right|$$

(3) TMH=PTMH/86

### 3.3. Criteria for model evaluation

The evaluation index is a key factor for measuring network performance, and tear meniscus segmentation is of practical significance only if the evaluation index meets the expectations (25). In the field of image segmentation, many evaluation indices can describe the network segmentation accuracy. Among them, the commonly used evaluation indicators are the intersection over union (IOU), dice coefficient, intra class coefficient (ICC) and sensitivity (26–28). We recorded the target region in label A1 and the prediction of the target region as A2.

(1)The IOU describes the ratio between the intersection and merging of the real and predicted results, and the closer the ratio is to 1, the higher the coincidence degree of the two. IOU is calculated as


(4) $$\text{IOU}=\frac{\mathrm{A1}\cap \mathrm{A2}}{\mathrm{A1}\cup \mathrm{A2}}$$

(2) The dice coefficient describes how similar the two samples are, and the closer the two samples are, the closer the dice coefficient is to 1. The dice coefficient is calculated as follows:


(5) $$D\,i\,c\,e=\frac{2\,\left|\mathrm{A1}\cap \mathrm{A2}\right|}{\left|\mathrm{A1}\right|+\left|\mathrm{A2}\right|}$$

(3) ICC = (variance of interest)/(total variance) = (variance of interest)/(variance of interest + unwanted variance), ICC can be used to evaluate the segmentation done by the models and the observers. I would like to use ICC to evaluate my model proposed in the paper compared with doctors in measuring TMH. The ICC ranges from 0 to 1, a high ICC close to 1 indicates high reliability of the model.

(4) In addition to the IOU and dice coefficient evaluation index, the network performance can be measured using sensitivity. TP: correctly predicted as tear meniscus/CCPR; FN: incorrectly predicted background as tear meniscus/CCPR; FP: incorrectly predicted as the background of tear meniscus/CCPR; TN: correctly predicted background.

Sensitivity refers to the ratio of the predicted correct region to the predicted total region in the prediction result, i.e., the accurate measurement of the network segmentation, and its calculation formula is as follows:


(6) $$S\,E=\frac{T\,P}{T\,P+F\,N}$$

### 3.4. Optimizer and learning rate updating strategy

The optimizer provides a direction for adjusting the neural network parameters in deep learning, which causes the loss function to approach the global minimum continuously and determine the global optimal solution. According to the different tasks, selecting the appropriate optimizer to optimize the parameters is necessary; otherwise, the loss function may remain in the local optimal solution, resulting in non-convergence of the network. In this study, the stochastic gradient descent algorithm (29) was used as the optimizer. The gradient is the vector pointing to the maximum value of the derivative of a function in the direction of a certain function at this point, that is, along this direction, the fastest change in the function value. Let the mean-square loss function be


(7) $$J(\theta \text{)}\;\text{=}\;\frac{1}{m}\sum_{i=1}^{m}{\left(x_{i}.\theta -y_{i}\right)}^{2},$$

where *θ*(*w*_1_,*w*_2_,*w*_3_,….,*w*_*n*_) is the weight vector, and *a partial derivative of each component* is determined using function *J*(*θ*) to obtain the gradient *g* = *J*′(*θ*); accordingly, the updated *θ* at the next moment is


(8) $$\theta_{\text{t}+1}=\theta_{\text{t}}-\alpha \cdot g,$$

whereθ_t_ is the last weight, θ_t+ 1_is the updated weight, andα is the learning rate that determines the step size for each parameter update.

The optimizer controls the direction of the parameter optimization update, whereas the learning rate controls the speed of the parameter optimization update. Generally, the learning rate decreases with the number of iterations. At the beginning of training the network, a larger learning rate can be set to allow the network to swiftly adapt to the training samples. When training to a certain extent, reducing the learning rate is necessary, which finely adjusts the network parameters and avoids the network from shaking. In this study, we used the cosine annealing (30) strategy to update the learning rate. The learning rate decreases in the form of a cosine function. According to the characteristics of the cosine function, learning first gradually decreases, subsequently accelerates the decline, and finally decreases slowly. The learning rate decay formula is


(9) $$L\,r_{t}=L\,r_{m\,i\,n}+\frac{1}{2}\,\left(L\,r_{m\,a\,x}-L\,r_{m\,i\,n}\right)\,\left(1+\text{cos}\,\left(\frac{N_{c\,u\,r}}{N_{m\,a\,x}}\,\pi \right)\right)$$

*Lr*_*t*_ refers to the current learning rate, *Lr*_*max*_ and *Lr_min_* refer to the maximum learning rate and minimum learning rate that we set in advance, and *N_cur_* and *N_max_* refer to the current iteration times and total iteration times, respectively.

## 4. Results

The designed deep convolutional neural network can accurately segment the tear meniscus region and CCPR in the ocular surface image, and the TMH can be evaluated using the segmentation results. First, the center of CCPR must be determined. To locate the center of the CCPR more accurately, we performed circular fitting and cavity filling of the CCPR followed by an external rectangle (actually a square). The center of the CCPR can be located by the pixel value and edge length of the left upper vertex of the rectangle. The upper and lower edges of the tear meniscus were obtained using edge detection.

### 4.1. Segmentation of target region

**FIGURE 5:**
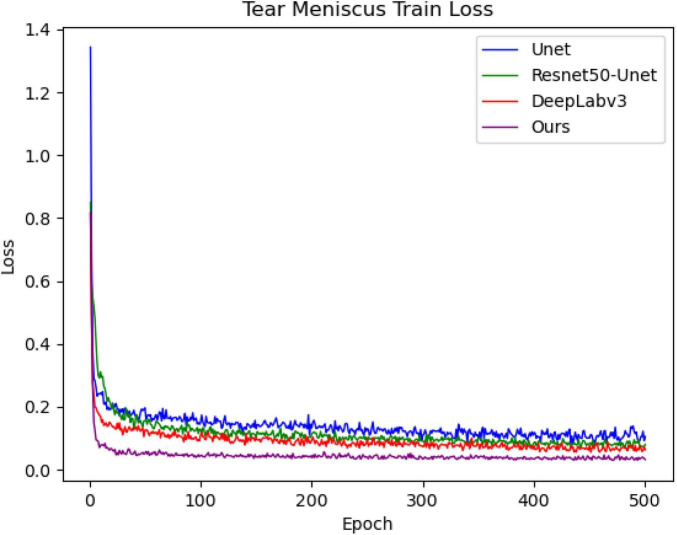
Loss of training process for tear meniscus segmentation by different models.

Each tear meniscus ocular surface image and its corresponding mask were uniformly cropped to 480 × 480, and subsequently, the image contrast was enhanced by the HSV random enhancement method. Data augmentation was achieved using rotation, translation, and inversion (31, 32) to improve the generalization ability of the model. Experimental hardware configuration during training and testing: Intel (R) Core (TM) i7-6700 CPU @ 3.40GHz, GPUNVIDIA GeForce RTX 1080. Experimental software configuration: The operating system was Windows10 with 64 bits, PyCharm Community Edition 2021.3, Python 3.6.13. All deep convolutional segmentation neural networks were set as follows: (1) SGD (momentum = 0.9, weight decay = 0.0001) was selected by the optimizer, and the learning rate was set as 0.0001. (2) The batch size was set to 4, and the maximum training epoch was set to 500. (3) A learning rate updating strategy was applied during the experiment. This strategy allows the learning rate to update every step instead of every epoch update, such that the network can be trained more effectively. After building the experimental environment and setting the initial learning parameters, different models are trained using the training set, and different models are used to segment the tear meniscus. The change in the loss value during the training process is shown in Figure 5. After training for 500 epochs for the training set, the loss value of the network built in this study decreased the fastest and dropped below 0.1.

**FIGURE 6:**
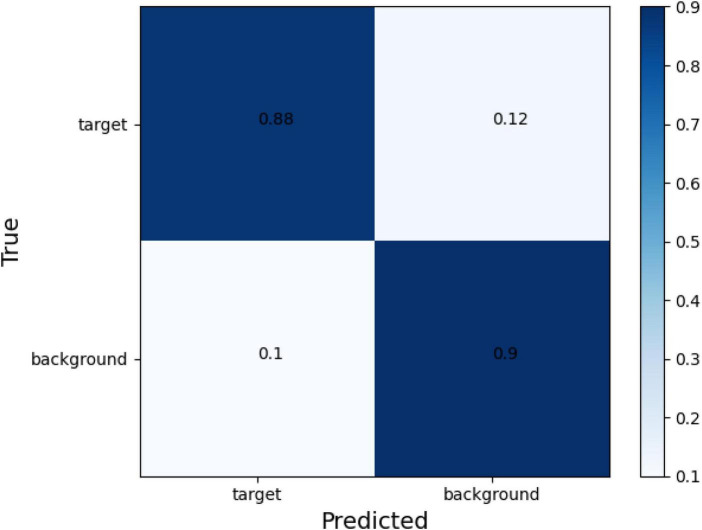
Confusion matrix of tear meniscus segmentation.

**FIGURE 7:**
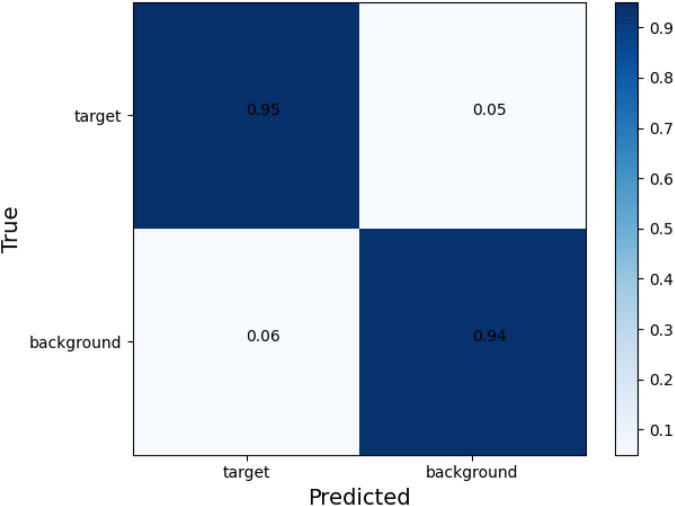
Confusion matrix of CCPR segmentation.

The confusion matrix of tear meniscus segmentation and CCPR segmentation used proposed network in this study showed in Figures 6, 7, respectively. To evaluate the segmentation performance of the deep convolutional segmentation neural network used in this study, we used the popular segmentation networks Unet, Resnet50-Unet, and DeepLabv3 to perform comparative experiments. In this study, the segmentation performance of different models for the tear meniscus region and CCPR were evaluated using IOU, dice coefficient, and sensitivity. The segmentation results of different models for the tear meniscus region are summarized in Table 1, with an average IOU of 0.896, dice coefficient of 0.884, and sensitivity of 0.887. The segmentation performance of our model was the best when the tear meniscus region was segmented. The segmentation results of the different models for the CCPR are summarized in Table 2, where the average IOU was 0.932, the dice coefficient was 0.926, and the sensitivity was 0.947. When the CCPR is segmented, the model segmentation effect is optimal. In summary, the proposed model can accurately segment the tear meniscus region and CCPR, which is conducive to the accurate measurement of the TMH.

**TABLE 1:** mIOU, dice coefficient, and sensitivity for tear meniscus region segmentation by different methods.

Method	mIou	Dice	Sensitivity
Unet	0.831	0.806	0.761
Resnet50-Unet	0.881	0.868	0.871
DeepLabv3	0.882	0.870	0.863
Ours	**0.896**	**0.884**	**0.877**

The bold values represent the best results for each metric.

**TABLE 2:** mIOU, dice coefficient, and sensitivity for CCPR region segmentation by different methods.

Method	mIou	Dice	Sensitivity
Unet	0.891	0.802	0.785
Resnet50-Unet	0.912	0.906	0.905
DeepLabv3	0.921	0.913	0.929
Ours	**0.932**	**0.926**	**0.947**

The bold values represent the best results for each metric.

### 4.2. TMH measurement

**FIGURE 8:**
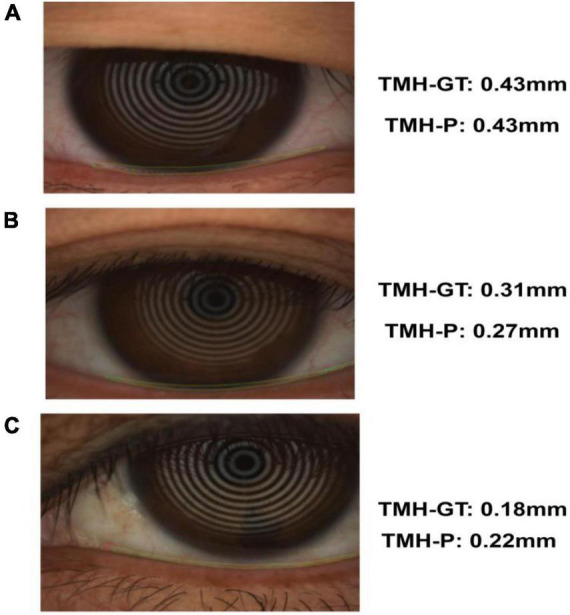
Example of tear meniscus segmentation; TMH-GT indicates the TMH of label, TMH-P indicates the TMH predicted in this paper: Panel **(A)** is accurate segmentation **(B)** is under segmentation **(C)** is over segmentation.

**FIGURE 9:**
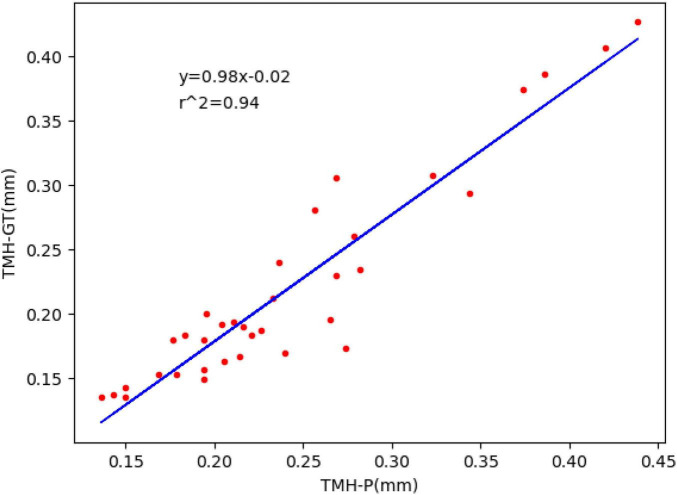
Fitted linear regression of true value and predicted value of TMH; ordinate represents the true value of TMH, abscissa represents the predicted value of TMH, and the blue line corresponds to regression line y=0.98x−0.02,r^2^=0.94.

The trained segmentation network combined with the image processing method was used to measure the TMH, and three ocular surface pictures in the testing set were selected, as shown in Figure 8, to show the prediction results of TMH. We outlined the labeled tear meniscus region and the predicted tear meniscus region with red lines and green lines, respectively. Figure 8A shows that the tear meniscus region is accurately segmented, and both the true and predicted value of TMH are 0.43 mm. Figure 8B shows that the tear meniscus region is under segmented, the true value of TMH is 0.31 mm, and the predicted value is 0.27 mm, which results in the predicted value of TMH being less than the true value. Figure 8C shows that the tear meniscus region is over segmented, the true value of TMH is 0.18 mm, and the predicted value is 0.22 mm, which results in the predicted value of TMH being greater than the true value. The true and predicted values in all testing sets were compared using linear regression, as shown in Figure 9, where the true and predicted values of TMH in all images in the testing set remained consistent, with a satisfactory regression line y0.98x−0.02, and the overall correlation coefficient was *r*^2^0.94. The ICC is used to evaluated the reliability of proposed model in the study, the ICC of TMH was 0.90, which showed good reliability.

## 5. Discussion

DED is one of the most common ocular diseases affecting visual function in 5–30% of the world’s population (33). DED causes a series of subjective symptoms and visual damage due to tear film instability accompanied by potential ocular surface damage. As the incidence of DED increases, it affects the visual quality of patients and thus affects their daily life; therefore, the evaluation of visual quality of DED patients has gradually received considerable attention. However, there are no uniform criteria for the diagnosis of DED, and fluorescein tear break-up time (34) and Schirmer test (35) are generally used to diagnose DED; however, these traditional diagnostic methods are invasive and unrepeatable (36–38). Studies have found that TMH is an important parameter of tear meniscus, and its value can be used to distinguish normal eyes from eyes affected by DED (39, 40); nonetheless, most of the measurements of TMH are manual or semi-automatic; for example, professionals are required to outline the upper and lower edges of the tear meniscus and select the measurement point of TMH, which is not only time consuming and laborious, but also the measured TMH is unrepeatable, which may lead to inaccurate diagnostic results. Therefore, it is highly important to design a fully automatic, noninvasive method for measuring TMH. Based on this, a method for measuring TMH is proposed in this study, in combination with deep learning and image processing methods. The acquisition of ocular surface images, detection of the tear meniscus region, and measurement of TMH are automatic and noninvasive. The prediction results of the TMH were consistent with the measurement results of professional doctors. The method proposed in this study can accurately measure TMH and can be used to assist doctors in the diagnosis of DED, which has important clinical and practical significance.

In this study, we first obtained the ocular surface image using K 5M equipment and eliminated the blurred image of the tear meniscus region caused by closing the eyes and not focusing during shooting. Subsequently, a deep convolutional neural network was built to segment the tear meniscus and CCPR regions. The segmentation network used in this study included two parts: feature extraction and image size restoration. The feature extraction part is composed of the adjusted Resnet50 and ASPP module in DeepLabv3. Segmented image size restoration was realized by bilinear interpolation sampling. In addition, an auxiliary output was elicited at the feature extraction stage by referring to the auxiliary output structure of GoogleNet and the output of the FCN. Finally, circular fitting was performed on the segmented CCPR to better locate its central point, and subsequently, the upper and lower edges of the tear meniscus were detected by edge detection to achieve the TMH, and after measuring the pixel values corresponding to the TMH, the final TMH values were obtained by 86 pixel/mm. The model used in this study exhibits an average IOU of 0.896, dice coefficient of 0.884, and sensitivity of 0.887 for the segmentation of the tear meniscus region and an average IOU of 0.932, dice coefficient of 0.926, and sensitivity of 0.947 for the segmentation of the CCPR region. The model built in this study can automatically identify and accurately segment the tear meniscus region and the CCPR region. A trained deep convolutional neural network was used to segment the ocular surface images in the testing set and predict TMH in combination with image processing methods; the regression line y=0.98x−0.02 (*r*^2^=0.94) was used to fit the true and predicted values of the TMH in the testing set.

The method proposed in this study for measuring TMH is advantageous because it is noninvasive and fully automatic. The ocular surface images used to assess the TMH were obtained by a professional K5M shooting instrument without touching the patient’s eyes throughout the procedure. Segmentation of the tear meniscus region and measurement of the TMH were achieved by a computer, the measurement method of TMH was easily implementable. Furthermore, the amount of calculation was small, and the physician was not mandated to select TMH measurement points, which eliminated the problem of inconsistent results owing to subjective assessments, reduced repeatability, and increased interobserver variability. The method proposed in this study can accurately measure TMH and assist doctors in DED screening.

The shortcomings of this study are that the number of datasets is small, and the quality is uneven, and certain images with eyes closed and blurred shooting are extant, necessitating continued collection of more high-quality images. The more datasets used to train the network, the more accurate the segmentation results of the network, such that a more accurate TMH is measured. As for segmentation network, with the deepening of the convolutional layer, the obtained feature map has a larger field of view, in which the shallow network focuses on texture features and the deep network focuses on the overall information of the picture. When pooling down sampling, it inevitably loses part of the edge information of the features, and this lost information cannot be recovered by upsampling alone, whereas the Unet network achieves the retrieval of edge features through the concatenation of features, which significantly improves the segmentation fineness. In the future, we can combine different networks, such as the DeepLab series and the Unet series, to improve and further improve the accuracy of segmentation.

## 6. Conclusion

In this paper, we propose a method to automatically measure TMH using deep learning combined with image processing. The measurement results of TMH obtained using the method proposed in this paper are consistent with clinical data, and this is clinically significant. In the future, with the continuous development and optimization of algorithms and the acquisition of more high-quality datasets, the accuracy of the measurement of TMH will increase, and it can be used to screen DED.

## Data availability statement

The raw data supporting the conclusions of this article will be made available by the authors, without undue reservation.

## Ethics statement

Ethical review and approval was not required for the study on human participants in accordance with the local legislation and the institutional requirements. Written informed consent from the patients was not required to participate in this study in accordance with the national legislation and the institutional requirements.

## Author contributions

CW and RH acquired, analyzed, discussed the data and drafted the manuscript. PG and PL analyzed and discussed the data. JW, WY, and XH acquired the clinical information and revised the manuscript. All authors contributed to the article and approved the submitted version.
